# Stakeholders' Perspectives on the Challenges of Emergency Obstetric Referrals and the Feasibility and Acceptability of an mHealth Intervention in Northern Iraq

**DOI:** 10.3389/fgwh.2021.662256

**Published:** 2021-05-26

**Authors:** Bridget Relyea, Alison Wringe, Osama Afaneh, Ioannis Malamas, Nicholas Teodoro, Mohammed Ghafour, Jennifer Scott

**Affiliations:** ^1^Women and Health Alliance International, Erbil, Iraq; ^2^Women and Health Alliance International, Paris, France; ^3^London School of Hygiene and Tropical Medicine, London, United Kingdom; ^4^École des hautes études en santé publique, Rennes, France; ^5^Beth Israel Deaconess Medical Center, Boston, MA, United States; ^6^Harvard Medical School, Boston, MA, United States

**Keywords:** maternal health, obstetric emergencies, mHealth, digital, humanitarian, feasibility & acceptability, conflict setting

## Abstract

The health system in northern Iraq has been weakened by conflict and the internal displacement of over three million people. Mobile phone-based interventions (mHealth) may improve maternal and neonatal health outcomes by enabling emergency referrals, facilitating communication between patients and providers, and improving patient data management; however, they have not been sufficiently studied in conflict-affected settings. We explored stakeholders' perspectives on challenges to obstetric referrals and the feasibility and acceptability of a mobile phone-based application to reduce delays in reaching emergency obstetric care in order to inform its development. We conducted a qualitative study in the Kurdistan region of northern Iraq from May to July, 2018. Using purposive sampling, we carried out 15 semi-structured interviews with coordination actors including healthcare management staff, government health officials, non-government health program managers and ambulance staff. The interviews explored obstetric care delivery, referral processes, mobile phone usage and mHealth implementation strategies. Eleven focus group discussions, which incorporated participatory activities on similar topics, were conducted with ambulance drivers, hospital and primary health center staff. Audio-recorded, transcribed and translated data were coded iteratively to identify emerging concepts, and analyzed thematically. Sixty-eight stakeholders (36 women and 32 men) participated. Challenges regarding the referral system included resource limitations, security concerns, costs and women's reluctance to be transported in male-staffed ambulances. In terms of obstetric care and decision-making, participants noted gaps in communication and coordination of services with the current paper-based system between health care providers, ambulance drivers, and hospital staff. Ambulance drivers reported incurring delays through lack of patient information, poor road conditions, and security issues. A prototype mobile phone application was found to be acceptable based on perceived usefulness to address some of the challenges to safe obstetric care and focused on phone usage, access to information, Global Positioning System (GPS), connectivity, cost, and user-friendliness. However, the feasibility of the innovation was considered in relation to implementation challenges that were identified, including poor connectivity, and digital literacy. Implementation of the app would need to account for the humanitarian context, cultural and gender norms regarding obstetric care, and would require substantial commitment and engagement from policymakers and practitioners.

## Introduction

Civil conflict in Iraq, beginning in 2014, has resulted in widespread internal displacement of the population, which was coupled by an influx of refugees from neighboring countries due to the crisis in Syria (1). Conflict and displacement have weakened the health system in Iraq through increased demand for services and a concomitant shortage of funding, health workers, supplies and equipment (2–4). The extent to which these shortages have impacted obstetric outcomes is difficult to measure since the most recent estimates of maternal and infant mortality were generated prior to the conflict (5–7). However, recent studies in the region have demonstrated that there are a high number of unplanned pregnancies, that antenatal care remains suboptimal (8) and due to barriers accessing health facilities, such as security concerns, over 10% of deliveries take place at home (9). Programs to improve healthcare coordination and coverage nationally, inclusive of displaced and refugee populations, have been implemented by the government and a number of non-governmental organizations (10, 11). Despite these efforts, coordinating emergency obstetric referrals and transfers between health care centers remains particularly challenging, and is likely to contribute to excess maternal mortality and morbidity in northern Iraq.

The “Three Delays Model,” developed by Thaddeus and Maine in 1994, is a conceptual framework for understanding delays in accessing and receiving emergency obstetric care. It has been applied in numerous studies, particularly in sub-Saharan African settings (9, 12–14), and to a lesser extent in conflict or post-conflict settings (15, 16). The model posits that maternal mortality is influenced by three delays: first, the delay in deciding to seek medical care for an obstetric emergency; second, the delay in reaching a health facility; and third, the delay in receiving adequate care once at the facility (17). This framework has been particularly effective in understanding and enabling the development of interventions to address obstetric delays in different settings, leading to reductions in maternal mortality and morbidity (18, 19). In terms of the second delay, effective interventions have included transportation vouchers, ambulances, communications equipment for health facilities and the construction of maternity waiting homes (20). Some authors have suggested the need to alter and expand the model to be more comprehensive of other factors such as gaps in referral systems, fuel for ambulances, ambulance waiting times and inter-facility communication delays, which were not suggested in Thaddeus and Maine's initial model (21). The conclusion of the aforementioned systematic review also proposes considering the individual experience through the Women's Health Empowerment Model (21).

In conflict-affected settings, addressing the second delay is particularly challenging due to insecurity and impeded access to health facilities. In such settings, there is an emerging body of evidence that mobile phone-based interventions (mHealth) can be used to deliver healthcare or health services (22, 23) thereby addressing some of the concerns related to insecurity. mHealth interventions have gained traction for improving health outcomes over the past decade, including those relating to maternal outcomes (24). Specific examples include mHealth interventions to strengthen healthcare coordination and delivery through preventative or educational tools for pregnant women, traditional birth attendants or midwives (19–23).

This qualitative study aimed to understand delays in emergency obstetric care in the study setting and the potential feasibility and acceptability for developing an mHealth intervention to address factors contributing to the second delay. Specifically, the aim of the innovation would be to coordinate referrals of internally displaced and refugee women living in northern Iraq by emergency response staff through transportation to appropriate level-of-care facilities.

## Materials and Methods

### Study Setting

We conducted a qualitative study in the Erbil and Dahuk Governorate of the Kurdistan Region of Iraq between May and July 2018. At the time of the study, the displacement camps in Erbil Governorate, located between 30 to 90 km from Erbil city center, ranged in size from 300 to 1500 families. In Dahuk Governorate, they ranged in size from 300 to 5000 families (25). Most of the camps had a fixed Primary Health Center (PHC) with a general practitioner or gynecologist and nurses or midwives, with staffing depending on the size of the facility. These facilities provided reproductive health services and some Basic Emergency Obstetric and Neonatal Care (BEmONC); however, few provided Comprehensive Emergency Obstetric and Neonatal Care (CEmONC).

### Participant Recruitment

To accomplish a purposive sample, we identified key stakeholders in the provision and coordination of emergency obstetric care and referrals in the region. These included healthcare workers, healthcare management staff, government health officials, non-governmental health program managers and emergency vehicle staff (Table 1). The field research team identified and contacted these individuals by email, phone, or in-person through the facilities where they worked. The research supervisor met with the management of health care facilities to introduce the study and obtain authorization for their staff to participate. Additionally, individuals who participated were asked to recommend other potential participants and thus snowball sampling was used to reach all participant groups and saturation. We determined saturation during the data collection phase when new data began to replicate previous findings and new information no longer emerged (26).

**Table 1:** Number of in-depth interviews and focus group discussions.

**Interview type**	**Respondent category**	**Number of interviews/groups**	**Number of respondents**	**Women**	**Men**
In-depth interview (IDI)	Emergency vehicle staff	5	5	0	5
	Hospital staff	0	0	0	0
	Public health care facility	1	1	1	0
	Coordination actor	9	9	3	6
**Total IDI**		**15**	**15**	**4**	**11**
Focus group discussion (FGD)	Emergency vehicle staff	2	10	2	8
	Hospital staff	5	23	13	10
	Public health care facility staff	4	20	17	3
	Coordination actor	0	0	0	0
**Total FGD**		**11**	**53**	**32**	**21**
**Grand total**		**26**	**68**	**36**	**32**

*Bold values are the sub-totals per interview type and the Grand total for both*.

### Data Collection

Data collection tools were developed by the research team based on similar studies in other geographic settings (27, 28) and using human-centered design concepts to ensure a participatory and iterative approach to the research (29). The human-centered design approach aimed to design and develop solutions with users and communities to first understand the problems that could be solved using innovative tools targeting feasibility, viability and desirability. Input was also sought from field workers based in Iraq on the relevance and suitability of the data collection tools for the study setting.

Semi-structured interview topic guides were developed by the research team composed of a research supervisor and two principal investigators with previous experience conducting research in humanitarian settings. Topic guides were developed for each participant sub-group in English, and then translated into Arabic and Kurdish (Sorani). Back translation was carried out for the Arabic translation to ensure that the original meaning had been retained, with corrections made where necessary, whereas the Kurdish translations were cross-checked by the two Kurdish speaking facilitators.

We conducted both in-depth interviews (IDIs) and focus group discussions (FGDs) (see Table 2). For IDIs, we selected coordination actors and emergency vehicle personnel, as this data collection approach was more suitable to the aims of the study and availability of participants. These semi-structured individual interviews focused on the coordination and delivery of antenatal health care services including obstetric referrals, challenges in ensuring obstetric referrals, means of work communication and mobile phone usage, and potential implementation strategies for a mobile phone-based app.

**Table 2:** Study Methods by participant group.

**Methods**	**Participant groups**	**Topic guide**
In-depth Interview	Emergency Vehicle Personnel; Public Health Care Facility Staff, Coordination Actor	Coordination and delivery of antenatal health care services including obstetric referrals, challenges in ensuring obstetric referrals, means of work communication and mobile phone usage, mobile phone app functions prototype and potential implementation strategies for a mobile phone-based app
Focus group discussion	Emergency Vehicles, Hospital staff, Public Health Care Facility Staff	Experiences of obstetric referrals and associated challenges; participatory learning activities including workday charting, referral decision making vignettes, and mobile phone app functions prototype

For FGDs, we selected personnel who mainly functioned in collaborative work settings. These included public health care and hospital staff (doctors, nurses, midwives, etc.), as well as emergency vehicle personnel. These semi-structured group interviews focused on collective experiences of obstetric referrals and associated challenges and participatory learning activities including workday charting to understand working patterns and practices. The groups were also given patient scenarios and asked to address these in order to better understand referral decision-making in real time. In addition, mobile phone application functionality options were presented visually with a prototype and discussed by the participants to document the preferences of intended users and acceptability and feasibility of an mHealth intervention. The authors define acceptability as the factors that affect the participants' willingness to use the application and feasibility of the implementation factors such as structural context, administration and capabilities, as derived from similar prior research (28, 30, 31).

In-depth interview and FGDs were conducted by three trained field workers and/or by the research supervisor in the language of preference for each participant (English, Kurdish or Arabic) in a private room in health care facilities run by non-governmental organizations (NGOs) in displacement camps, and in maternity hospitals and NGO organization headquarters. One interview was conducted by Skype by a trained Arabic-speaking research assistant to accommodate the availability of the participant.

The facilitators (two men and one woman) were hired specifically for this research based on their previous experience working in conflict-affected settings and populations in Iraq. None of the participants were known to the facilitators prior to data collection, however participants may have been familiar with the NGO coordinating the study. The facilitators completed a one-day training on data collection ethics and tools, the informed consent process, and practiced facilitation and interview techniques through role play. During data collection, the facilitators provided information sheets to participants on the purpose, foreseeable risks and benefits and the voluntary nature of the study, and obtained signed informed consent. Key demographic and contextual information were collected prior to the interviews and focus groups, however no identifiable information was collected. Focus group discussion participants were requested to maintain the confidentiality and anonymity of the other participants. Memos were written by notetakers and interviewers directly following the interviews and groups. Audio files were transcribed and translated into English by a local Arabic/Kurdish speaking translator. The research supervisor was responsible for quality control during data collection and was present for selected interviews, coordinated post-interview debriefing sessions, and reviewed audio recordings and memos (in English). Discussions were held remotely with the principal investigators periodically throughout the data collection period.

### Data Analysis

English transcripts and field worker memos were coded manually and iteratively in Word documents and an Excel spreadsheet. The coding framework was designed based on the topic guides used during data collection. Thematic analysis was carried out by two individuals through familiarization with the data, generating initial codes, searching for themes among the codes, reviewing the themes and incorporating them into a write up of the analysis (32). Once an initial set of FGDs and IDIs were analyzed, the coding framework was discussed and jointly refined by the co-investigators before being applied to the remaining ones (see Table 3). Reflexivity was considered through analyzing rival explanations and presenting cases/findings that did not present the main pattern of findings. Our findings were also triangulated by employing different types of data collection methods (interviews and participatory focus groups) with a diverse group of participants and stakeholders.

**Table 3:** Key themes and codes.

**Key themes**	**Respondent group contributions**	**Related codes**
Reasons for delays in accessing emergency obstetric care	All respondents	- Accessing care—Security/corruption - Accessing care—Road conditions - Difficulty finding location - No female nursing staff for ambulances - Knowing when to deliver - Delivery in hospital - Reasons for not seeking care/home birth - Low educational attainment - Insufficient resources - Quality of care
Obstetric care and referral decision making process	All respondents	- Urgency of referral (time) - Decision for referral - Transportation for referrals - Referral communication - Referral information - Important considerations for referral
Acceptability and feasibility of mHealth solutions	All respondents	- Potential of the App—Yes - Potential of the App—No Connectivity/internet issues - Type of electronics - Mobile phone/internet usage - Useful information/functions of app
Implementation challenges	All respondents	- Digital literacy and training - Stakeholder buy-in/engagement - Connectivity/internet issues

### Ethical Approval

We received approval for this study by both Beth Israel Deaconess Medical Center Institutional Review Board (IRB Protocol number: 2018P000219) and the General Directorate of Health (dated 02/05/2018 and reference number 2718). Given the nature of collecting data in a humanitarian setting (33–35), additional effort was made to ensure that participants' rights were maintained throughout the recruitment process, informed consent process, interviews and that the data were securely stored and transferred. The research team were familiar with the relevant ethical considerations and security issues in this particular context.

## Results

In total, 15 respondents participated in the interviews and 53 in focus groups. Refusal to participate was noted by three potential participants, including two coordination actors and one ambulance driver for reasons related to unavailability to attend a focus group discussion. The mean age of participants was 45 years for those participating in the IDIs and 34 years for those in the FGDs (Table 1). The majority of ambulance drivers were men, whereas the majority of nurses, midwives and doctors were women, reflecting typical gender distribution for these roles in this region.

### Reasons for Delays in Reaching Emergency Obstetric Care From Displacement Camps

A number of sub-themes were identified highlighting reasons for delays including the current referral system, resource limitations, security context, and health care human resources. Participants described how the referral system in place between health facilities and maternity hospitals in city centers was used on an *ad-hoc* basis and was primarily paper-based and phone call-based. In the northern Governorate of Dahuk, there was an emergency response number that centralized the ambulance response service. However, in other regions, calls for referrals and ambulance requests were not systematized and required health workers in facilities to have knowledge of ambulance drivers' mobile phone numbers. Due to the distance from most of the camps to the city center (over 30 km), there were some ambulances stationed at health facilities in the camps so as to be more readily accessible in emergencies.

Several referral challenges were identified and related to resource limitations for delivering emergency obstetric care including transportation costs, equipment availability, quality of care in health facilities, distance to health facilities, road conditions, security and corruption.

Limited available resources were described as directly impacting the quality and level of care that could be provided. For example, more than one patient would be delivered in a single ambulance if only one vehicle was available. Ambulance drivers explained that they would sometimes need to take two or even three patients in the same vehicle. In areas where the Islamic State was present, ambulances were often destroyed, which significantly reduced the number available. In addition to a shortage in ambulances, there were often fuel shortages for vehicles. This could render a vehicle unusable and unable to transport a patient when needed. In some cases, as an alternative, pregnant women called upon private taxis to bring them to maternity hospitals particularly as internally displaced persons (IDP) tended not to own or have private vehicles available to them.

In terms of the security context, continued insecurity in the region meant that there were movement restrictions that impacted the delivery of health services, including the transportation of patients. Roads were often backed up due to security checkpoints for crossing into the urban areas where hospitals were located, preventing ambulances from being able to reach their destination in a timely way. Needing to pass multiple checkpoints could lead to long delays in reaching health facilities:

…*when you refer such a case and in front of you [are] a lot of check points, sometimes the patient needs [to wait] three to four hours.* – Coordination actor, male, IDI participant

Study participants also indicated that narrow road infrastructure and traffic jams contributed to delays in reaching some patients. This was further compounded by the fact that locations were not always clearly specified, as one participant explained:

*A lot of times, an ambulance would go somewhere but wouldn't find the location. And this is a problem for us, because it would have to go from this street to that one and it gets late until it finally finds it. This issue wastes too much of our time.* - Coordination actor, male, IDI participant

Costs were also reported as a prohibitive factor. Participants described multiple points during the referral process and the provision of care more generally when patients were faced with expected and unexpected costs. Although the ambulance transportation service was free of charge, pregnant women had other costs as described by a female health worker below, and also faced the transportation cost of returning to the camp after giving birth or receiving care.

*Both transportation and the cost are obstacles. There are some cases that need to be referred, for example, those who have [had] previous Cesarean sections or twin pregnancies, they absolutely need to be referred, so, before the delivery, they have to be fully checked at the checkpoints and the [Security and Intelligence] forces ask them to pay money and also when they arrive at the hospital, they need to pay for the hospital too.* - Health worker, female, FGD participant

In addition to the structural challenges impacting delays, ambulance drivers noted challenges relating to the provision of care for pregnant women whilst being transported. Despite most participants being strongly motivated to help patients to the best of their abilities, they often felt constrained by staff shortages and cultural norms that required pregnant women to be accompanied by a family member or another woman:

*Our problem is when I go to refer a case of a pregnant woman, I must have a female nurse with me. Otherwise, what can a man do for a woman?* – Ambulance driver, male, IDI participant

*Among the big problems we have, the first one is the quality of our ambulances…Having medical tools and specialized equipment inside the ambulance is very necessary. Another big problem, about which the DoH has always been supportive, is that we have a lack of staff, especially the female staff which is very necessary for our society.* – Coordination Actor, male, IDI participant

However, in practice, ambulance drivers, who were exclusively men in this study setting, were rarely accompanied by female medical staff due to a lack of available personnel. Some described how this could impact the decision of women and their families to use an ambulance for an emergency referral:

*Our biggest problem is when we go to refer a pregnant woman without having a female nurse with us. People still call us, but there are some women who [are] shy to go with a man nurse, because they are afraid of the possibility of delivering in the ambulance mid-way, and this actually has happened with our colleagues where the patient has given birth in the ambulance. What can the poor man do for her in the ambulance? She is a woman and she needs a woman to be with her. It is very necessary to have a female staff specifically for pregnant cases...* - Ambulance driver, male, IDI participant

Other participants noted that in some cases, nurses from the primary health center in the camp or family members accompanied the patient; however, this was not done systematically. Additionally, female patients were unable to be accompanied by their male partners due to them being denied entry to the city center when passing security checkpoints.

At the receiving hospital, resource limitation and overcrowding continued to be a recurrent theme. One hospital staff member explained:

*There is an overburden on the hospital because of the camps, as you know how our circumstances have been in the last four years.* – Hospital staff, FGD participant

Coordination mechanisms were put into place to manage the increased burden and influx of patients in hospital. One staff member at a maternity hospital explained:

*They were transporting the patients and the coordination was very good. Although the patients were facing challenges, they still managed to reach out to us. And we, as the hospital, managed to make it through all the obstacles and loads. We helped them as much as we could despite the bad economic and financial conditions. We also had a lack of medicine and medical equipment and therefore the load was too big on the Kurdistan Region.* – Coordination actor, female, IDI participant

It was also noted that cases that were not emergencies or not IDPs would arrive at the hospital themselves or by a minibus service provided from the camps.

### Healthcare Workers' Obstetric Care and Referral Decision Process

The findings on referral decision-making were based primarily on consensus that emerged during the FGDs. The decisions regarding whom, when and where to refer were made by doctors at the primary health centers in the displacement camps based on certain criteria. Formal checklists were not used to support decision-making, yet factors that were considered included medical history, capacity of the facility to provide appropriate care, timing of labor and available transportation. Patient preferences could be considered, but were not determining factors:

*They (the patient or family) can choose if they go to Erbil or to Mosul…Depending on what the financial situation [of] their family situation is…They can certainly ask to be referred and they might give their reasons but at the end of the day the doctor makes that decision about how, who would be referred and it needs to have a medical or a mental health reason*. – Hospital staff, FGD participant

One hospital staff employee highlighted how delays in these decisions could often influence patient outcomes:

*Time is very important. Sometimes, in just 5 min, because of a simple delay, a case might go out of our hands*. – Hospital staff, FGD participant

As such, determining the urgency of referral was considered critical. Information about the receiving hospital, including the availability of care, was also considered to be a particularly important factor in referring patients:

*The risk level, right, this is her risk level. But then what is left is the transportation, details about the receiving hospital so that I would know whether it is available or not and not randomly refer the patient there without being sure.* – Primary health center worker, FGD participant

Additionally, health workers at the hospitals indicated that receiving more information on medical records and on any care already provided would be useful to have communicated from the referring facility. In some cases, healthcare providers at primary health care facilities near or in camps indicated that they would follow up with the hospital on their patients that were sent for emergency care, whereas others hoped to have news upon their return to the camp. On the other hand, focus group participants generally agreed that for both acute and less acute emergencies, important factors included the availability of translation services, the cost of care, and patient preference. Overall, health care workers and hospital staff determined their work satisfaction and success based on the health outcomes of their patients and response to treatment, which may also inform their care provision and decision-making process.

*But the best time for me is when I deliver a baby and the baby comes out safe and the mother is safe without having any bleedings or problems, that's when I feel very happy.* – Primary health center worker, FGD participant

### Feasibility and Acceptability of mHealth Solutions to Reduce Delays in Reaching Facilities

Following discussions on the current referral system and the related challenges, the potential for introducing mHealth was explored during the IDIs and FGDs. Most participants indicated that they used their mobile phones for personal purposes only, except for some hospital staff that used them to search for medical references. Respondents felt that they often did not have time to use their phones while at work and others raised safety concerns around using an mHealth solution while driving.

*I am on my way and I receive a phone call, I will take a quick look at it but will not answer unless it is from our team or else I will lose focus and, God forbid, an accident might happen. This is why I don't use it.* – Ambulance driver, male, IDI participant

Literacy and digital literacy were raised as potential barriers for using a mobile phone-based app to facilitate referrals, with some ambulance drivers reporting they were unaware of how to use smartphones and indicating that they only used the calling function on their phones for work purposes. Some expressed openness to the introduction of technology but felt that the current paper system was better.

*Communication between the staff, doctors and the administration is mostly done through mobile [phone calling].* – Coordination Actor, female, IDI participant

Nonetheless, the majority of participants suggested that a tool to assist with emergency obstetric patient referrals would be useful if it facilitated their work, enhanced communication and ensured that pregnant women were able to access adequate care in a timely manner. There was a general willingness to test out new solutions to potentially provide better care and a desire to see improved outcomes for their patients.

*I think they will support it because it is a new system and it will help and facilitate serving the patient and it will also make the work easier for the medical staff and enable them to reach the patient immediately.* – Coordination Actor, female, IDI participant

Some respondents indicated a sense of urgency to implement a proposed digital solution to address the challenges that had been discussed. Some felt that the use of technology would improve the patient experience, yet they also felt that this improvement may go unnoticed by patients.

In terms of specific functions, ambulance drivers and health workers noted that a mapping or Global Positioning System (GPS) function would be useful to decrease delays in reaching their patient for pick up and arriving at the referral hospital. They also felt that a digital technology could support their work in providing continuity of care:

*Actually, it will be very useful because it will allow you to follow up on the patient by calling the receiving doctor and knowing that the patient has arrived at a certain place in a short time faster than you could ever call the ambulance.* – Primary health care worker, FGD participant

Participants felt strongly that improving the communication between facilities and during transportation, through a calling function, would improve the quality of care through efficiently and accurately estimating the time of arrival, patient location and directions. Feedback and coordination between the health care providers to close the referral loop was also considered to be a useful component of a potential mobile phone application.

*It will be useful if it is not going to be used in only one place because when you use the application, the other side also should use it so that there will be coordination. You cannot get benefit from it if you use it alone.* – Hospital staff, FGD participant

Designated communication channels and facilitation of exchanges between facilities were considered to be important traits of a mobile phone-based app for referrals. The mobile phone application that was tested during the focus group discussion was based on a flow of communication from the provider in a PHC making the decision to refer, inputting patient data, and then on to the ambulance driver who would receive a notification requesting transportation for a patient. Hospital staff confirmed that it would be important to have patients' medical histories including their name, age, gravida, parity, medications, previous surgeries, as well as care and any medications received to date. A digitalised system was seen as more efficient in comparison to the current paper-based and call-based system.

### Potential Implementation Challenges for a Mobile Phone-Based Application for Referrals

The primary barriers to using mobile phones in this setting included issues with connectivity and network availability. Some respondents indicated that mobile phone communication units were generally affordable; however, others felt that it was very expensive on a daily and weekly basis to purchase units for an internet connection. It was also indicated that there was limited connection in certain areas, especially in displacement camps and on roads. In more remote areas, further away from city centers, these issues, along with erratic electricity supplies were considered particularly important:

*We have neither service coverage, nor an internet connection. Or we cannot recharge our phones because our electricity rationing is so bad. We need electricity*. Primary health care worker, FGD participant

Especially in hospital settings, where there was access to free Wi-Fi services, ambulance drivers felt that using technology and a mobile application could positively affect their work and the coordination of referral services. However, in settings without Wi-Fi or with limited or slow connectivity, some respondents suggested that any digital tools would only work if they were available or functional in an “offline” mode. A majority of participants indicated they had their own smartphones and were able to use the different functionalities, though digital literacy was a concern for some, especially older participants who had not previously used mobile phone applications. Some study participants emphasized the importance of the tool being user-friendly with illustrative icons and a guided flow of use.

In terms of integrating a mobile phone app into their current workflow, some participants felt that it would add additional burden to their work and some ambulance drivers considered it could be a distraction while driving:

*The difficulties are that I have too much work to do and there is a big pressure on me, I can neither answer the phone nor make a phone call.* – Ambulance Driver, FGD participant

Data security was also cited as a concern by several participants who proposed that different levels of access and sign-in techniques for the app would be necessary. Suggestions were also made that this could be used for more appropriate case management archiving. Sustainability and long-term implementation of the mobile phone-based application was also recognized as critical to its effectiveness, including the need for training and piloting. It was suggested that all actors be required to engage in the development and roll out in order to facilitate its long-term use. Adherence by healthcare workers and ambulance drivers would need to be supported by the government and funders. Finally, for scaling up and equity of access, stakeholders felt that the application should be used among both displaced populations and host communities as there would be benefits that could serve both populations.

## Discussion

This qualitative study conducted among key stakeholders identified challenges related to the provision and coordination of emergency obstetric care in conflict-affected northern Iraq and identified opportunities for an mHealth intervention. Barriers that contributed to the second delay (i.e., the delay in accessing a health facility) in this context included communication challenges between ambulance drivers and health facilities, security issues, and the cost of referrals and care for patients. The lack of female health workers in ambulances was also highlighted as a barrier to seek care, as well as impacting the quality of care that could be provided for pregnant women during transfers. Other studies have also shown similar factors impacting the second delay (20, 36), including one qualitative study on maternity services in the Iraqi context, which demonstrated safety issues, limited resources and maternity services constraints (37).

Our study focused on addressing the second delay in accessing obstetric care from the supply side perspective, which has been studied extensively in sub-Saharan African settings (38), but to a lesser extent in the conflict affected regions in the Middle East. Our findings suggest that there is potential to develop the agency and involvement of ambulance personnel in the referral process and in the design of tools to improve the effectiveness of transportation and communication procedures and to provide decision-making support. However, the female patient's perspective and decision-making process related to accessing care is important when considering mitigation strategies to the three delay model (21), even though it may be difficult to address with mHealth. Further studies on the patient perspective could explore potential adaptations to the mHealth intervention that may address these concerns, including for example, patient preferences for female health workers to be present during referrals.

Our findings also demonstrated that a mobile phone-based app would be acceptable among a range of stakeholders as an approach to improve the effectiveness of obstetric referrals. Study participants were willing to accept an mHealth intervention based on the perceived usefulness of the technology meaning that this tool could potentially improve their workflow and enhance job performance. This related to the participants' sense of work satisfaction and success, which was reported as a patient responding well to treatment and both mother and baby being safe after delivery. Other studies have also found communication systems (SMS, phone calls) as an effective method to introduce mHealth technology to health care workers (30), including using prototypes of the technology during focus group sessions (39). While improving communication systems can be addressed with an mHealth intervention, other aspects of the healthcare system, such as resource availability, infrastructure and potential security issues, persist, though are certainly necessary components of addressing delays (23).

Research in other settings has demonstrated a reduction in maternal deaths based on reducing the second delay by using mHealth tools to improve communication and coordination (24), including text-messaging based tools tested in Rwanda to register pregnancies and request ambulances (40). However, as in our study, the Rwandan research also found that the effectiveness of mHealth tools may be limited if other barriers to care, such as the availability of ambulances being in service, is not addressed (40). As such, informing the context adaptation necessary for an mHealth solution is critical for implementation, as is understanding what can and cannot be addressed through a mHealth intervention.

Beyond the intuitive appeal for some, capacity to use the new technology, also referred to as digital literacy, emerged as one concern that could undermine uptake of an app for improving referrals in this setting, particularly among ambulance drivers. Additionally, there would be an advantage to healthcare workers being able to use their own mobile devices or potentially be in a setting to receive one for work purposes. This is particular relevant as there is lower mobile device ownership among women. To address infrastructure limitations and connectivity concerns in rural and remote areas, developing a mobile app with offline capabilities would be beneficial. In relation to mHealth interventions, Drury et al. created a model for their implementation in resource-limited settings based on five components that need to be considered including context, content, connectivity, capacity and community (41). A synergy between these components needs to be achieved for an investment in mHealth to achieve its full potential benefits (41).

Although our study focused on the second delay attributed to maternal mortality, it is important to note that the intricate links between the three delays require approaches that address the other delays if obstetric outcomes are to be improved. A revised prototype of the application was subsequently tested during a pilot stage, yet not reported on in the scope of this research. Hence, the implications of the research and further testing could demonstrate the potential for mHealth to improve the effectiveness of health care staff in conflict-affected settings as well as enhance the capacity of those involved in the referral process, especially ambulance drivers. Other mHealth research among community health workers has suggested similar implications particularly with regards to creating better tools and workflow systems that enabled them to make fewer human errors, better decision making and rapid response (42).

There are several limitations to this research that need to be taken into consideration when interpreting our results. Firstly, given the qualitative nature of the study, social desirability bias may have influenced participants' responses, although this should be partially mitigated by triangulating the responses from different participant groups and through using both interviews and group discussions. Furthermore, the background of the researchers, including the interviewers, and some language barriers where participants did not fluently speak all the same language (Kurdish or Arabic) may have influenced the findings. As interviews with pregnant or postnatal women were not conducted, we were not able to consider their experiences or perceptions of barriers to obstetric referrals. However, our focus on the provider perspective was driven by our aim of assessing how well an app could address the barriers to the provision of emergency obstetric care, including referrals. Further research in this setting could include a component to capture women's perspectives on accessing obstetric care and referral challenges and the potential of mHealth to address these concerns. Additionally, further research should consider looking into digital technology usage by female patients for obstetric emergencies, as well as the use of human-centered design approaches in humanitarian contexts more generally. Finally, due to specific conditions in our study setting, some of the findings from this research may not be applicable elsewhere.

One particular strength of our study was its focus on factors underlying the second delay to accessing obstetric care in Iraq. Furthermore, the use of an innovative, human-centered design approach (25, 43) to garner insights on solutions to mitigate causes for referral delays in northern Iraq enabled us to ensure that further development of the prototyped application would better meet users' needs. The collaborative and participatory approach is possible to replicate in other low-resource or conflict-affected settings as a way to engage end-users in the design and solution-development process to facilitate locally-driven innovation and ownership.

In conclusion, we found several challenges that contributed to the second delay in accessing obstetric care in northern Iraq, where conflict and displacement have burdened the health system. A mobile phone-based app to organize and coordinate referrals was deemed acceptable by the stakeholders in this setting and has potential to reduce delays relating to transportation. The acceptability of the proposed intervention was aided by a willingness to adopt technology in order to improve the current system. Nevertheless, implementation challenges include internet connectivity, low digital literacy skills and resource shortages. In order to address these potential bottlenecks to its implementation and uptake, ongoing support from policymakers and other actors involved in the coordination of maternal health services as well as sustained funding for training, scale-up and regular updates to the app would be needed. The findings of this research may also guide development and piloting of other mHealth interventions in similar settings.

## Data Availability Statement

The raw data supporting the conclusions of this article will be made available by the authors, upon reasonable request and without undue reservation.

## Ethics Statement

The study involving human participants was reviewed and approved by the Beth Israel Deaconess Medical Center Institutional Review Board and the Directorate of Health based in Erbil. The patients/participants provided their written informed consent to participate in this study.

## Author Contributions

BR, JS, AW, OA, and IM: conception and design. BR, JS, AW, IM, and MG: administrative support. BR, JS, AW, and OA: provision of study materials or patients. BR, JS, AW, OA, IM, and MG: collection and assembly of data. BR, JS, and NT: data analysis and interpretation. All authors manuscript writing and final approval of manuscript.

## Conflict of Interest

The authors declare that the research was conducted in the absence of any commercial or financial relationships that could be construed as a potential conflict of interest.
