# Supplementary material for: Stakeholders' Perspectives on the Challenges of Emergency Obstetric Referrals and the Feasibility and Acceptability of an mHealth Intervention in Northern Iraq
Source: Front Glob Womens Health. 2021 May 26;2:662256. doi: 10.3389/fgwh.2021.662256 (PMC8594019; doi:10.3389/fgwh.2021.662256)
Supplement: Supplementary file 1 [file Data_Sheet_1.PDF]

In-depth Interview – Emergency Vehicles Personnel: Topic Guide

Welcome and Introduction

Hello, my name is _________ (name of interviewer). I am from _________________ (name of partner organization, e.g. Women and Health Alliance International), working in collaboration with Harvard Medical School in Boston, Massachusetts, United States of America. We are working on a research study about the use of phones and phone apps to facilitate access to maternal health care in this area of Iraq. The results of this study will inform the development of a mobile phone application used by health care providers.

You have been selected to participate in this study because you work with a facility/MDU/ emergency vehicle that is linked to ______________ (name of partner organization; WAHA, DoH, etc.) and is of interest to the our research project of developing a mobile phone application to improve maternal health in Northern Iraq.

Guidelines

There are no right or wrong answers. We will be recording the conversation, however your name and identifying information will not be shared with anyone beyond the study team or be published.

We will start by asking you some general questions about your background and experience providing maternal health care.

Section 1: Background and Experience

1. Can you tell me about yourself? What is your current role? How long have you been in this role?
    a. Can you please describe your main tasks and responsibilities?
    b. Can you tell me briefly about any other roles that you had prior to this?

c. Can you tell me about your qualifications and training that you have had for undertaking your current role? *Probe: educational background, on-the-job training etc.*
d. What type of services do you provide?
e. Can you describe to me what you do in a typical day at work? *Probe: What do you do when you arrive at work? And then? etc*
f. Who do you provide services to? *Probe: Local communities? Internally displaced persons? Refugees? Where are they from?*

<u>Section 2: Service Delivery Information</u>

2. In your opinion, what are the main obstacles faced by pregnant women in seeking and receiving medical attention? Especially for internally displaced persons? For refugees?
    a. How are decisions made at the household level to seek medical care? *Probe: Finances? Need/condition of the person? Distance to appropriate care? Means of transport? Beliefs/attitudes towards health facilities? Who makes the decision?*
    b. What are the obstacles in receiving care at the health center level? *Probe: in terms of distribution of resources? Human resources? Available medications and medical equipment? What is the chain of command for decision-making?*
    c. What are the obstacles at the health systems level? *Probe: District-level systems, national policies? Can you provide an example?*
    d. How do you contribute to this process? *Probe: providing care, providing transport, ensuring essential medications, facilitating referrals, etc.*
3. Based on your professional experience, where do most deliveries usually take place?: home? Health clinic? Hospital? What do you think are the main factors driving this? *Probes: cultural issues, practical issues, health systems issues, etc.*
4. How often are deliveries attended by a medical professional? Can you explain why or why not? *Probe: Can you provide an example?*
5. What are normally the series of events at the time of labor for women? *Probe: In internally displaced persons camps? In refugee camps?*

   a. What are normally the series of events at the time of labor in an active conflict setting? *Probe: where do the delays occur? Delay in decision to seek care? Delay in reaching care, accessing transportation? Delay in receiving quality care, inadequate facilities or referral systems*

6. How do you manage patients in need of emergency obstetric care*, can you provide a recent example?*
   a. Can you describe in detail this process? *Probe: communicating, admitting, triaging, service delivery and/or discharging the patient.*
   b. Are your vehicles used for other purposes besides patient transportation and health care provision? *Probe: examples and frequency, and duration*
7. How big is the geographical area that your ambulance/MDU provide services to? How long does it take on average to reach a patient that needs care?
8. In general what are the available means of transport for transfers?
   a. In emergency cases?
   b. In less urgent cases? *Probe: Ambulances, private cars, public transport*
   c. What are the challenges faced when referring cases? *Probe: lack of vehicles? Quality/maintenance/fuel for vehicles? Poor communication? Lack of referral mechanisms in place? Quality of roads? Security of roads?*
9. Who is involved in making a decision to refer pregnant women between facilities? Who is a key player in that decision? *Probe: Pregnant woman, husband/family member, health care worker (doctor/nurse), emergency transportation personnel, hospital management? etc?*
   a. What are the main drivers for a referral decision? *Probe: resources available, case complication, expertise available in each facility?*
   b. What are the main challenges and breaks in the chain of referrals? What is breaking and why?
10. When a primary health care center refers a pregnant woman to a hospital, what information is typically provided about the case? What information is ideally provided?

    a. What information is typically received at the hospital? What information is ideally received at the hospital?

11. How do health care workers communicate with each other to inform each other about those referrals? *Probe: written referral letters, emails, SMS, phone calls, etc.*
12. What information about patients is transferred between facilities? *Probe: Patient medical files?* How are you involved in this process? What type of information do you need to complete your job of transportation/MDU services?
    a. How are those information transferred? *Probe: In person, by the transport (driver), online, other? Please explain.*
13. What are the means of communication between health care facilities and your vehicle for patient referrals? Who is usually in charge of communication at your vehicle? *Probe: Mobile calls, call center, SMS*

We will now ask you some general questions about your mobile phone use.

Section 3: Mobile Phone Usage

14. Do you use communication devices on a regular basis for personal and/or professional purposes? If so, which ones? *Probe: Mobile phones, computers, laptops, tablets, pagers, others? Is it mainly for personal or professional use?*
15. [If participant reports using a phone]: When using your mobile phone, which specific services and features do you use? Normal calling feature, texting features and/or mobile phone applications? *Probe: Chat, social networks, Skype, Google maps, transportation apps, agenda and scheduling functions health or wellness apps, etc. Can you name a few?*
    a. Can you do this independently or with help of another person? If so, who? *Probe: Family member, neighbour, colleague, etc.*
16. Can you tell me about the last time you used one of the above mentioned devices for professional purposes related to your work? *Probe: When? What did you use it for and in what circumstances?*

   a. Can you give me any examples of how using a mobile phone has been helpful in your work?

17. What reasons are there for not using mobile devices? What difficulties do you face when using your mobile device? Do you have coverage in all of the places where you work/live/commute? *Probe: Camp settings? Other difficulties? Charging batteries?*
18. How much does it cost to own a phone? What about internet connection packages (3G)? Does your phone have? Who pays for these costs? *Probe: Is it easy and/or inexpensive to top-up your phone? How often do you pay for top ups? Does your employer cover a portion of the costs?*
19. Do you think that a mobile application to facilitate communication among health care workers, health centers, MDUs and ambulances would be helpful to improve maternal health care service delivery? *Probe: In what ways? Who should be involved in this type of mobile application communication? If not, why not? If mentions challenges, probe how these may be overcome?*

Now we will ask some specific questions to help us develop a mobile phone application to improve maternal health care service delivery.

<u>Section 4: Parameters and Functionality</u>

20. Mobile applications for health typically involve one or multiple main functions; in your opinion what are some of the main functions to improve maternal health care service delivery?
    Probe: If he/she has no answer, could propose the following functions: data collection and management; health care decision support and guidelines; communication between providers; referrals between facilities; triage of cases; others…
21. What are in your opinion the features that could be included in the proposed application? *Probe: text messaging between providers; chatting options; data sharing; lab results transfer; medical record transfer; voicemail systems; patient identification options; GPS localization of MDUs, patients or available services; others…*

22. In your opinion, what is the most important information concerning the patient that should be collected and transferred through the application? *Probe*: *basic information, needed care, case urgency, medical history, etc.*
23. When we talk about "app interfaces", we mean how apps look and function on the screen from the user end. If an app was developed to aid referrals of pregnant women, what kind of interface do you think would make it most appealing for users? What are your suggestions? *Probe: Symbols, words, colors*
24. Do you prefer a personal sign-in or a general sign-in per facility? Why?

Now we are going to talk about some possible ways that we might disseminate and use a mobile phone application for communication between health care providers and facilities regarding case management and referrals.

<u>Section 5: Dissemination and Uptake Strategies</u>

25. From a health coordination perspective, in which ways do you think the usage of an app for healthcare workers to refer pregnant women between health facilities would impact health care delivery positively or negatively? *Probes:*
    a. How do you think it might facilitate access to health care? How do you think it might improve health care delivery? Why or why not?
    b. How do you think it might facilitate referrals between health facilities? *Probe: Triaging important cases? Time saving? Facilitating medical records transfer?*
    c. *Any possible negative impacts? Lack of up-to-date information, etc.*
26. Do you think that there would be support or resistance to introducing this technology into the health system generally, and health facilities specifically? *Probe: How might this technology be introduced into existing work and decision- making policies? Types of training…*

27. How do you think this technology would impact patient experience and information security? Can you provide an example? *Probe: shared facility phones, expectations around patient/provider interaction, monitoring of phone use, etc.*
28. How acceptable do you think the app would be to health care workers and mobile units personnel? Would you use it yourself if it was available today?
    a. How acceptable do you think the app would be to community members and patients?

Please feel free to share any remaining ideas or reflections about the questions mentioned.

<u>Section 6: Wrap-Up</u>

29. How do you see technology and the use of mobile phone apps for supporting health being incorporated into daily health services in the next 5-10 years?
30. Do you have any other feedback or advice for the developers of this mobile app technology?
31. Are there any other issues or ideas that you would like to bring up? Any suggestions or feedback?

Thank you for your thoughts and time.
