# Supplementary material for: Stakeholders' Perspectives on the Challenges of Emergency Obstetric Referrals and the Feasibility and Acceptability of an mHealth Intervention in Northern Iraq
Source: Front Glob Womens Health. 2021 May 26;2:662256. doi: 10.3389/fgwh.2021.662256 (PMC8594019; doi:10.3389/fgwh.2021.662256)
Supplement: Supplementary file 2 [file Data_Sheet_2.PDF]

In-depth Interview – Coordination Actors: Topic Guide

Welcome and Introduction

Hello, my name is _________ (name of interviewer). I am from _________________ (name of partner organization, e.g. Women and Health Alliance), working in collaboration with Harvard Medical School in Boston, Massachusetts, United States of America. We are working on a research study about the use of mobile phones and phone apps to facilitate access to maternal health care in this area of Iraq. The results of this study will inform the development of a mobile phone application used by health care providers.

You have been selected to participate in this study because your role as a coordination actor in health care services in the area where this research is conducted.

Guidelines

There are no right or wrong answers. We will be recording the conversation, however your name and identifying information will not be shared with anyone beyond the study team or be published.

We will start by asking you some general questions about your background and experience providing maternal health care.

Section 1: Background and Experience

1. What type of organization and department do you work with? *Probe: The Department of Health, United Nations Population Fund (UNFPA), UN High Commission for Refugees (UNHCR)?*
2. What is your role with health service coordination? How long have you been in this role? Can you describe your main tasks and responsibilities?
3. Can you tell me briefly about any other roles that you had prior to this? *Probe: Roughly how long in each role?*

4. Can you tell me about your qualifications and training that you have had for undertaking your current role? *Probe: educational background, on-the-job training etc.*

Section 2: Health Care Coordination

5. What type of health care facilities does your organization or department work with? What type of services do these facilities provide? Whom do they provide those services to? *Probe: Local communities? Internally displaced persons? Refugees? Where are they from?*
6. How do health facilities monitor and evaluate their activities? *Probe: Paper tracking, mobile devices, monthly meetings?* How well does this system work?
    a. How do you as a coordinating actor measure success of health care facilities services? *Probe: Number of cases? Number of lives saved?*
    b. What do you feel needs to be improved in term of health service delivery and monitoring? *Probe: What are the biggest challenges in terms of service delivery?*
7. What are the processes for enacting changes in health service delivery?
    a. Can you describe your role in decision-making processes in terms of changes in the way health services are delivered?
8. How would a new tool be maintained? By whom? *Probe: health care workers, nurses, doctors, hospital managers, government?*

Now I will ask you some questions on health service delivery for pregnant women.

Section 3: Health Service Delivery

9. In your opinion, what are the main obstacles that pregnant women face in seeking and receiving medical attention? Especially for internally displaced persons? For refugees?
    a. How are decisions made at the household level to seek medical care? *Probe: Finances? Need/condition of the person? Distance to appropriate care? Means of transport? Beliefs/attitudes towards health facilities? Who makes the decision?*

    b. What are the obstacles at the health center level? *Probe: in terms of distribution of resources? Human resources? Available medications and medical equipment? What is the chain of command for decision-making?*
    c. What are the obstacles at the health systems level? *Probe: The District-level systems, national policies? Can you provide an example?*
10. How does your organization or department contribute and influence this decision-making process?
11. Based on your professional experience, where do most deliveries usually take place?: home? Health clinic? Hospital? What do you think are the main factors driving this? *Probes: cultural issues, practical issues, health systems issues…*
12. How often are deliveries attended by a medical professional? Can you explain why or why not? *Probe: Can you provide an example?*
13. What are normally the series of events at the time of labor? *Probe: In internally displaced persons camps? In refugee camps?*
    a. What are normally the series of events at the time of labor in an active conflict setting? *Probe: where do the delays occur? Delay in decision to seek care? Delay in reaching care, accessing transportation? Delay in receiving quality care, inadequate facilities or referral systems?*
14. When a health care facility receives a pregnant woman for the first time, what information is typically gathered? How is this information collected? What information should ideally be gathered?
15. Who is involved in making a decision to refer pregnant women between facilities? Who are the key players in that decision? *Probe: Pregnant woman, husband/family member, health care worker (doctor/nurse), emergency transportation personnel, hospital management? etc?*
    a. What are the main drivers for a referral decision? *Probe: resources available, case complication, expertise available in each facility?*
    b. What is the process of deciding for the course of medical care offered?

16. What are the available means of transport for those transfers? Probe: *Ambulances, private cars, public transport, patient manages own transport*
    a. How is this decided? *Probe: Urgency of care? Availability of transport? Cost? Other reasons?*
17. Are you aware of the services provided by mobile health units? Do you know patients who have used them? How do you coordinate your services with those of mobile health units or emergency transport? *Probe: Phone calls? Online system? Word of mouth?*
18. When a health care facility receives or refers a pregnant woman to another health care facility in another location, what information should typically/ideally be sent with the woman? What information should typically/ideally received at the other facility?
    a. How are patient medical records transferred between facilities? *Probe: In person, by the transport (driver), online, other? Please explain.*
19. What are the means of communication between facilities used for referrals? *Probe: written referral letters, emails, SMS, phone calls, etc.*
20. What are the challenges faced when referring cases? *Probe: lack of transportation? Poor communication? Lack of referral mechanisms in place? Wait times at referral facility? Why?*

We will now ask you some general questions about means of communication and mobile phone use.

<u>Section 4: Means of communication and Mobile Phone Usage</u>

21. What type of devices are available for personal and/or professional use to health care workers in your region? *Mobile phones, computers, laptops, tablets, pagers, others?*
22. Do you think that mobile phones are used by health care workers for professional reasons? How? What do they use it for?
    Probe: Chat, social networks, Skype, Google maps, transportation apps, agenda and scheduling functions health or wellness apps, etc. Can you name a few?

a. How easy do you feel that mobile phone applications are to use? What makes them easy or not to use?

23. In general, what reasons are there for not using mobile devices? What difficulties do you face when using your mobile device? Do they have coverage in all of the places where you work/live/commute? *Probe: Camp settings? Other difficulties? Charging batteries?*
24. How much does it cost to own a phone? What about a 3G connection service? Who pays for these costs? *Probe: Is it easy and/or inexpensive to top-up your phone? How are top ups paid for? Do employers normally cover a portion of the costs?*
25. Do you think that a mobile application to facilitate communication among health care workers, health centers, MDUs and ambulances would be helpful to improve maternal health care service delivery? *Probe: In what ways? Who should be involved in this type of mobile application communication? If not, why not? If mentions challenges, probe how these may be overcome?*

Now we will ask some specific questions to help us develop a mobile phone application to improve maternal health care service delivery.

<u>Section 5: Parameters and Functionality</u>

26. During your work day, what functions of a mobile application would be most useful to improve maternal health care service delivery? *Probe: Data collection and management, monitoring and evaluation, Health care decision support and guideline, Communication between providers, Referrals between facilities, Triage of case, Other?*
27. What are in your opinion the features that could be included in the proposed application? *Probe: Messaging between health providers/facilities, Sharing patient data, Transferring lab results/medical records, Phone/Voice communications, Patient identification options, GPS localization of MDUs, patients or available services, Other.*
28. In your opinion, what is the most important information concerning the patient that should be collected and transferred through an application? *Probe: basic information, needed care, case urgency, medical history, etc.*

  a. *How is this data collected and monitored? Who is involved in this process? How can it be improved using an app?*

29. When we talk about "app interfaces", we mean how apps look and function on the screen from the user end. If an app was developed to aid referrals of pregnant women, what kind of interface do you think would make it most appealing for users? What are your suggestions? *Probe: Symbols, words, colors. Can you provide an example of a communication app interface that you like?*

Now we are going to talk about some possible ways that we might disseminate and maintain a mobile phone application for communication between health care providers and facilities regarding case management and referrals.

<u>Section 6: Dissemination and Uptake Strategies</u>

30. From a health coordination perspective, in which ways do you think the usage of an app for Health care workers to refer pregnant women between health facilities would impact health care delivery positively or negatively? *Probes:*
    a. *Facilitate access to health care? Facilitate care and improve health care delivery? Why or why not?*
    b. *Facilitate referrals between health facilities? Probe: Triaging important cases? Time saving? Facilitating medical records transfer?*
    c. *Any possible negative impacts? Lack of up-to-date information, etc.*
31. Do you think that there would be support or resistance to introducing this technology into the health system generally, and health facilities specifically? *Probe: How might this technology be introduced into existing work and decision- making policies?*
32. How do you think that this technology would impact patient experience and information security? Can you provide an example. *Probe: shared facility phones, expectations around patient-provider interaction, monitoring of phone use, etc.*
33. Which types of training do you think are most effective in transmitting information to health care providers?

34. How acceptable do you think the app would be to HCW?
    d. How acceptable do you think the app would be to community members and patients?
    e. Do you think that the app would face any issues relating to sustainability in the long term?
    f. How do you envision coordination actors being involved in disseminating the application?
    g. How do you see your involvement in supporting, and maintaining it? And using the data that could be collected through the app for monitoring and evaluation purposes?

Please feel free to share any remaining ideas or reflections about the questions mentioned.

Section 7: Wrap-Up

35. How do you see technology and the use of mobile phone apps for supporting health being incorporated into daily health services in the next 5-10 years?
36. Do you have any other feedback or advice for the developers of this mobile app technology?
37. Are there any other issues or ideas that you would like to bring up? Any suggestions or feedback?

Thank you for your thoughts and time.
