# Supplementary material for: Stakeholders' Perspectives on the Challenges of Emergency Obstetric Referrals and the Feasibility and Acceptability of an mHealth Intervention in Northern Iraq
Source: Front Glob Womens Health. 2021 May 26;2:662256. doi: 10.3389/fgwh.2021.662256 (PMC8594019; doi:10.3389/fgwh.2021.662256)
Supplement: Supplementary file 3 [file Data_Sheet_3.PDF]

Focus Group Discussion Guide – Medical and Para-Medical Health Care Workers – HCWs

Site: ____

Name of facilitator: ___________________________

Name of note taker: __________________________

Date: _______________________________________

Starting time of session: ______________________

Ending time of session: ________________________

Stakeholder group *(please choose one)*:

☐ Public Health Facility Staff;

Name of facility: _________________________

☐ Hospital Staff;

Hospital name: __________________________

☐ MDUs

☐ Ambulance personnel

If applicable; subcategory: ______________________

Probe: doctors, nurses, paramedics, drivers, etc.

Participant	Age	Gender	Marital Status	Occupation/Role	Working facility
Participant 1					
Participant 2					
Participant 3					
Participant 4					
Participant 5					
Participant 6					

Welcome and Introduction

Hello, my name is _________ (name of interviewer/facilitator). I am from _________________ (name of partner organization, e.g. Women and Health Alliance International). We are working on a research study about the use of phones and phone apps to facilitate access to maternal health care in this area of Iraq. The results of this study will inform the development of a mobile phone application used by health care providers.

We are conducting interviews and focus group discussions. This group has been invited to participate in this study because of you work in the facility/vehicle with which______________ (name of partner organization; WAHA, DoH, etc.) is providing health services.

Guidelines

There are no right or wrong answers, only differing points of view.

Brief open-ended questions will be asked in addition to group participatory activities that will be conducted The focus group will take approximately 60-90 minutes.

The focus group will be recorded using an audio-recorder. Neither your names nor any identifying information will be linked to the recording. After we transcribe the discussion, we will destroy the recordings. To facilitate understanding, please have only one person speaking at a time. You don't need to agree with others, but you must listen respectfully as others share their views.

Rules for cellular phones and pagers if applicable.

We will start with a group activity where you will get to graph the workload level in a typical day at work.

Section 1: General Information and Population Health Care Needs

1. What type of health care services are provided at your facilities? Who are those services provided for? What are their most common health care needs?

2. How about maternal health care and delivery services? Are those services available? By which facilities? How would you evaluate the quality of care provided?
3. PLA: A typical day: (15 mins)

   (Facilitator instructions: distribute needed materials: timeline sheet to each participant with pens, markers, sticky notes, etc) See example and blank timeline.

   Explain to participants that the x-axis is the timeline, starting with 8am to 6pm, and that the higher the graph is, the more workload there is during work. They closer it is to x-axis, the less work they got. Keep the attached example in mind while you explain to participant what is expected of them to draw the graph.

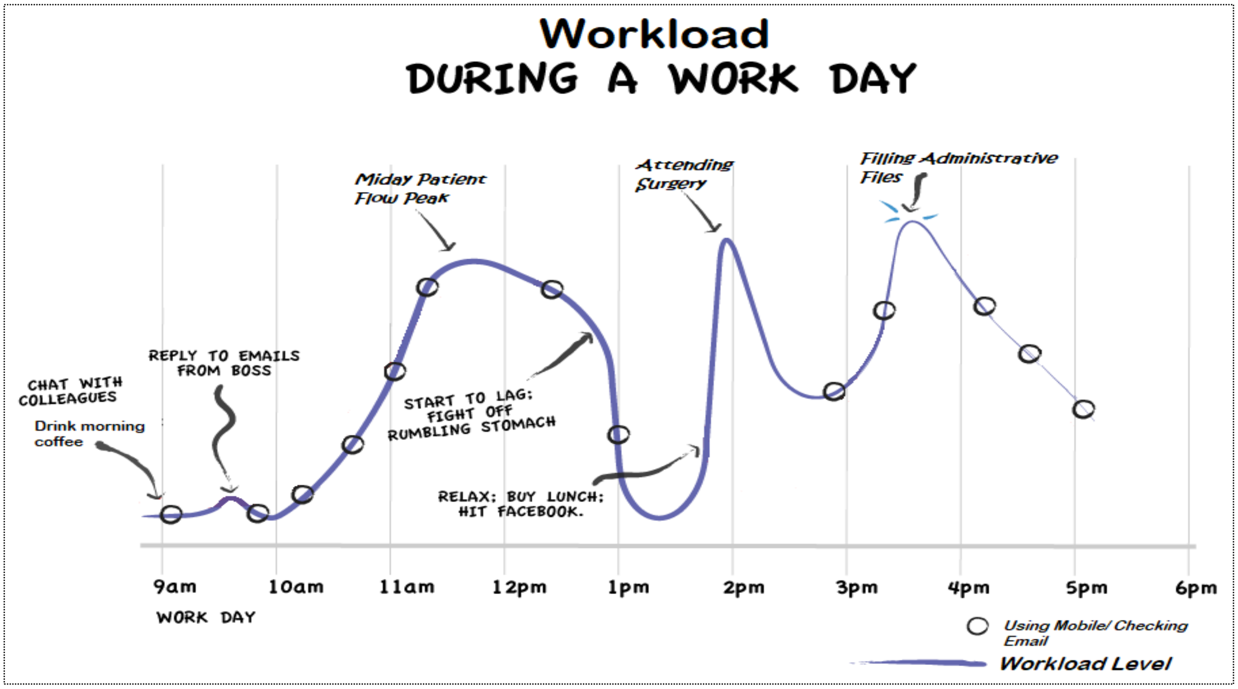

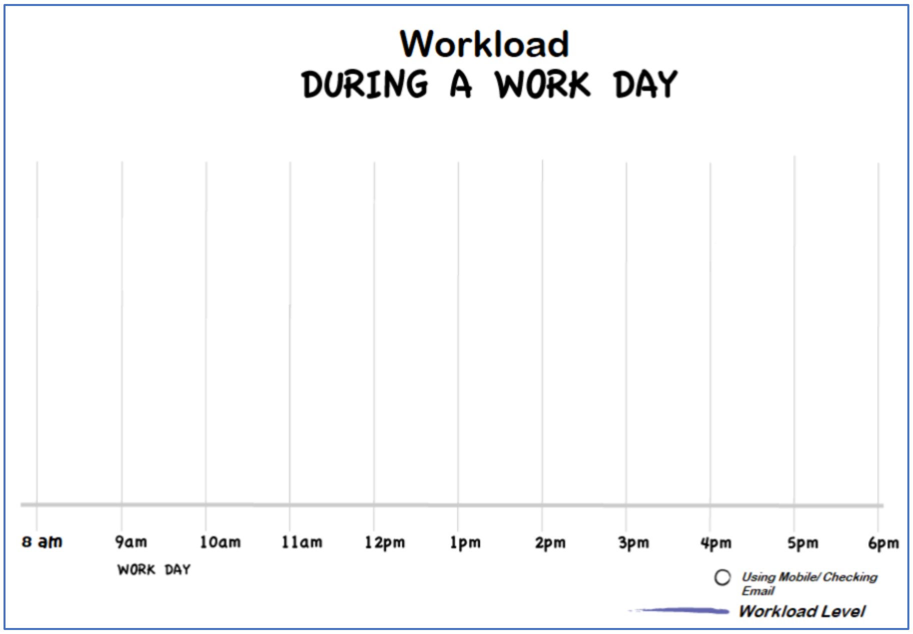

You have 5 mins to draw your workload yesterday using the timeline. After the 5 mins we will ask questions and have a group discussion around this activity.

Probe: Suggest the following elements to include on the timeline:

- *Labeling instances where they feel workload is high, overwhelming moments, etc.*
- *Labeling instances where they feel workload is low, they have time for themselves, or have time to help others*
- *Labeling feelings and reflections about certain moments during the day.*
- *Instances where individuals use their mobile phones or electronic devices (could include as a circle on the graph).*

Now that you have finished your timelines, I will ask you few questions about the work that you do and your workday:

a. Looking at your timeline, what do you like about your job? Where do you feel most satisfied? How do you define success in your workday?
b. Looking at your timeline, what are the most challenging moments in your workday? *Probe: is it at a certain time of the day? Once a particular situation arises? With specific cases? Can you provide example?*

c. How do you collaborate with your colleagues? In which situations do you need help from you colleagues? *Probe: When does this happens (eg. I need to do this but can't so I need other to do xxx) providing a helping hand when you have the time? Or ask of help when needed? Can you provide an example when collaboration works or doesn't work?*

Now we are going to ask you a few questions about how you use your mobile phone:

4. How do you contact/communicate with your colleagues? How do you contact/communicate with people in other health care facilities?
   Probe: Available channels of communication between HCWs: face-to-face meetings, written notes for colleagues, voicemail, telephone, SMS texting, etc.
5. Which of the following devices do you use on regular basis? *Mobile phones, computers, laptops, tablets, pagers, others?*
6. When using your mobile phone, which specific services and features do you use? Normal calling feature, texting features and/or mobile phone applications? *Probe: Chat, social networks, Skype, Google maps, transportation apps, agenda and scheduling functions health or wellness apps, etc. Can you name a few?*
7. What reasons are there for not using mobile devices? What difficulties do you face when using your mobile device? Do you have coverage in all of the places where you work/live/commute? *Probe: Camp settings? Other difficulties? Charging batteries?*
8. How much does it cost to own a phone? What about internet connection packages (3G)? Does your phone have? Who pays for these costs? *Probe: Is it easy and/or inexpensive to top-up your phone? How often do you pay for top ups? Does your employer cover a portion of the costs?*
9. Is mobile phone used for work purposes? How and in what circumstances? *Probe: communication with other healthcare workers? With management staff? With other facilities for referrals? For education purposes for clinical procedures?*
   a. Is it acceptable to use mobile phone for personal reasons during working hours?

b. Can you give me any examples of how using a mobile phone has been helpful in your work?

10. Does your work provide free, available, reliable internet connection to be used for work purposes? *(Wi-Fi, DSL, line connection, etc.)*

Now we will move on to the discussion and ask some questions about pregnant women's health care needs.

<u>Section 2: Service Delivery Information</u>

8. Based on your professional experience, where do most deliveries usually take place?: home? Health clinic? Hospital? What do you think are the main factors driving this? *Probes: cultural issues, practical issues, health systems issues…*
9. How often are deliveries attended by a medical professional? Can you explain why or why not? *Probe: Can you provide an example?*
10. What are normally the series of events at the time of labor? *Probe: In internally displaced persons camps? In refugee camps? In an active conflict setting?*
11. In your opinion, what are the main obstacles that pregnant women face in seeking medical attention? Especially for internally displaced persons? For Refugees? *Probe: Deciding to seek care? Reaching care? Receiving quality care? Knowledge or information about care options? Transportation? Costs? Availability of services/providers?*

<u>For Hospital and PHC personnel:</u>

a. When receiving a pregnant woman for the first time, what are typically/ideally the information to be gathered? What is the process of deciding for the course of medical care offered?

b. If you were receiving a pregnant woman in your health facility that had been referred to you by another health worker from another location, what information would you ideally hope that the woman arrives with?
c. When you are referring a pregnant woman to another facility, what are the information that you typically/ideally send with her?
d. What are the referral mechanisms available between facilities? How do health care workers from different facilities communicate and share information? *Probe: written referral letters, emails, SMS, phone calls, word of mouth etc.*

<u>*For Ambulance/MDU personnel:*</u>

a. How are you involved in the referral process of a pregnant women between facilities? What type of information do you need to complete your job of transportation/MDU services?
b. What are the means of communication between health care facilities and your vehicle for referrals? Who is usually responsible for communication within your unit? *Probe: Mobile calls, call center, SMS*

<u>*For all participants (Hospital, PHC, Ambulance and MDU personnel)*</u>

12. Who is involved in making a decision to refer pregnant women between facilities? Who are the key players in that decision? *Probe: Pregnant woman, husband/family member, health care worker (doctor/nurse), emergency transportation personnel, hospital management? Etc.?*
    a. What are the main drivers for a referral decision? *Probe: resources available, case complication, expertise available in each facility?*
    b. What is the process of deciding for the course of medical care offered?
13. What are the available means of transport for those transfers? Probe: *Ambulances, private cars, public transport, patient manages own transport*
    a. How is this decided? *Probe: Urgency of care? Availability of transport? Cost? Other reasons?*

14. How are patient medical records transferred between facilities? *Probe: In person, by the transport (driver), online, other? Please explain.*
15. Are you aware of the services provided by mobile health units? How do you coordinate your services with those of mobile health units or emergency transports? *Probe: Phone calls? Online system? Word of mouth?*
16. Do you think that a mobile application to facilitate communication among health care workers, health centers, MDUs and ambulances would be helpful to improve maternal health care service delivery? *Probe: In what ways? Who should be involved in this type of mobile application communication? If not, why not? If mentions challenges, probe how these may be overcome*

Section 3: Vignettes and Card Ranking Exercise. *Only for PHC personnel (otherwise skip to section 4)*.

Now we will conduct a short 15-20 mins exercise to reflect on what we have spoken about so far.

We will introduce 3 scenarios describing cases of pregnant women that are seeking care at your facility. I will read the scenarios out loud and then ask you to rank the cards and put them in groups according to your perceived importance when making a decision of referral for each patient scenario. There will be three groups according to importance: "Highly Important" (max 3 cards), "moderately important" and "Not Important". You have roughly 5-10 minutes to complete the ranking in a descending order of importance in each group of all scenarios.
Facilitator instructions: Start with the first scenario, read it out loud to the group, and then pass the scenario card around for them to see it. Then place the ranking cards on a central place (Ex. A table) where everyone can participate. Ask them to choose the three most important cards and place them in order in the "Highly Important" group then ask them to sort the rest of the cards in either group "Moderately Important" and "Not Important" with descending order of importance in each group.

The following scenario cases are to be handed out:

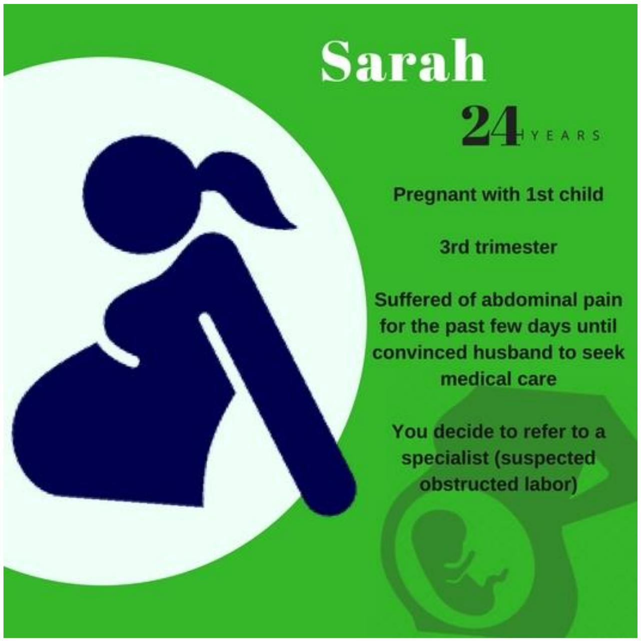

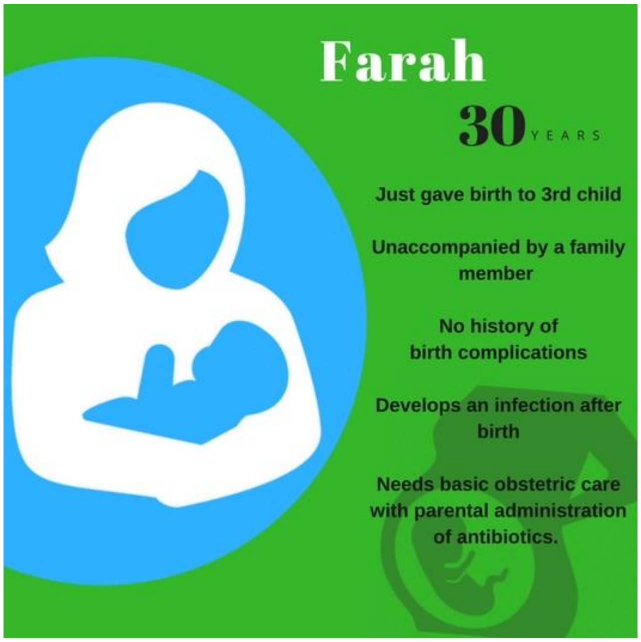

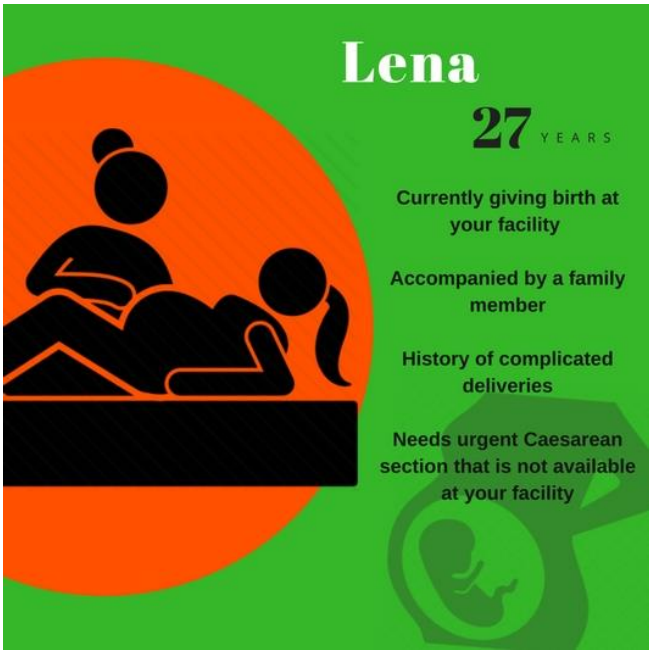

The following ranking cards are to be handed out:

Type of facility (MDU, hospital, Secondary PHC)
Distance, ETA, Wait time

Available Staff (Midwives, OB-GYN, Nurse, etc.)
Route (security/safety)
Facility management (NGO/DoH)
Capacity of services offered
Patient history (previous visits – min info input)
Location of patient community/home
Provider relationship/familiarity with facility<br>*Availability of ambulance*
Patient preference (cost, language, culture)
Medical translation service available
History of closing the loop (confirming arrival)
Community/culture of hospital (surroundings)
Rating of hospital (quality of care)
Cost of care (cost of recovery time or medicines)

Facilitator Instructions: for each scenario separately, ask the following questions, and allow time for discussion before moving on to the next:

a. Why did you rank the cards in this way? What are the factors in the case that influenced your decision? *Probe: mother age, severity of case, family accompanied, etc.*
b. Can you explain the choice of the "Highly Important" group (first three cards), and why you feel they are the most important? Can you explain the ranking of those cards?
c. Can you explain the ranking of the cards put in the group "Moderately Important" in a descending order? What makes them of less importance than the previous group?
d. Can you explain the choice of the "Not Important" group list of cards? What makes them not important in this scenario?

Facilitator instructions: Ask participants to look at the three scenarios which will be placed next to each other in the middle. Ask the following questions:

e. Are the three cards placed in the “Highly Important” group similar or different across different scenarios? Why are they similar or different? Are there cards that has always been grouped as “Highly Important”?
f. Are there any cards that were not important in some of the scenarios and became of importance in others? What makes them important in this case?
g. Are there cards that are “Not Important” across all scenarios? Why so?
h. What is/are the most influencing variables in the scenarios that affect the decision making process?
i. What information that are not provided in the scenarios that would have helped you to make a referral decision if was available? *Probe: different contexts? Other patient or facility variables?*
j. What other scenarios that you could think of, that might result in a different ranking of the cards and possibly affect the decision making process when making a referral?

Now we will speak about your expectations and opinions to help us develop a mobile phone application to improve maternal health care service delivery. We have developed a prototype of the proposed application that we would like you to test out. We will try it out together and then discuss your feedback and comments.

<u>Section 5: Parameters and Functionality</u>

17. <u>PLA: Mobile App Sacrificiel Concepts: (15 mins)</u>
Facilitator Instructions: Pass around the device with the prototyped application and let people try it out, observing their reactions and capturing their comments and feedback. Introduce a context where participants could imagine themselves in a health care facility and a pregnant woman arrives, needing a referral for an emergency caesarian section and they need to use the app to refer and/or receive her referral to the health care facility where they work. Then ask the following questions:
An example of what the prototype app looks like:

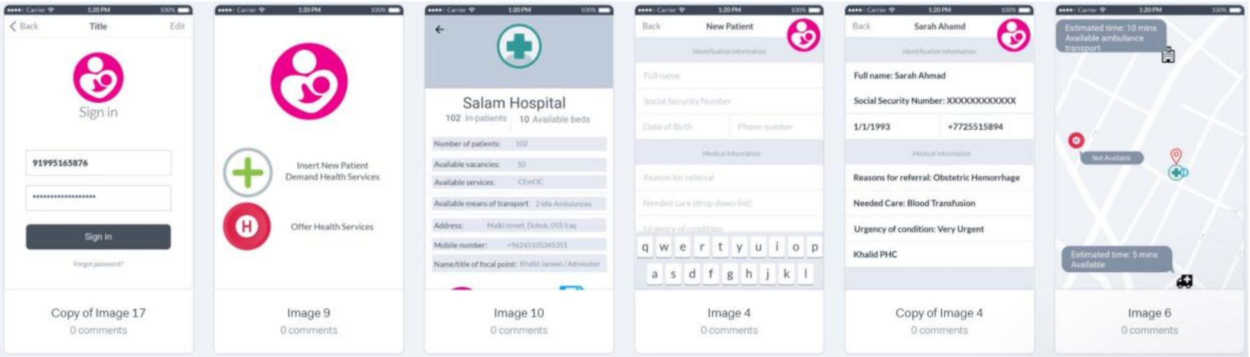

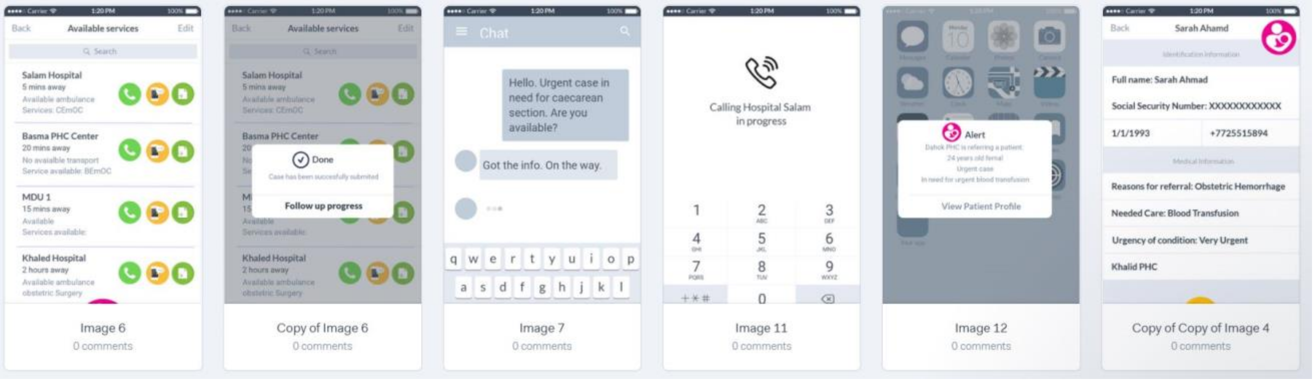

a. Does anyone have general feedback and comments about the application?

b. What did you like most about the prototyped application? What did you like least?

c. Do you find it easy to use?

d. Did you prefer the interactive map or the list of available services? Why? Which one do you think would be more efficient?

e. Did you prefer a personal sign-in or a general sign-in per facility? Why?

f. When we talk about "app interfaces", we mean how apps look and function on the screen from the user end. What kind of interface do you think would make it most appealing for users ? What are your suggestions? *Probe: Symbols, words, colors*

18. Do you use communication devices on a regular basis for personal and/or professional purposes? If so, which ones? *Probe: Mobile phones, computers, laptops, tablets, pagers, others? Is it mainly for personal or professional use?*

19. [If participant reports using a phone]: When using your mobile phone, which specific services and features do you use? Normal calling feature, texting features and/or mobile phone applications? *Probe: Chat, social networks, Skype, Google maps, transportation*

apps, agenda and scheduling functions health or wellness apps, etc. Can you name a few?

   a. Can you do this independently or with help of another person? If so, who? *Probe: Family member, neighbour, colleague, etc.*

20. Can you tell me about the last time you used one of the above mentioned devices for professional purposes related to your work? *Probe: When? What did you use it for and in what circumstances?*
21. In general, mobile applications for health typically involve one or multiple main functions; in your opinion what are the most useful main functions to improve maternal health care service delivery? *Probe: [If there are no answers, could propose the following functions]: Data collection and management, Health care decision support and guideline, Communication between providers, Referrals between facilities, Triage of case, Other?*
22. What are in your opinion the features that could be included in the proposed application: *Probe*: *Messaging between health providers/facilities, Sharing patient data, Transferring lab results/medical records, Phone/Voice communications, Patient identification options, GPS localization of MDUs, patients or available services, Other.*
23. In your opinion, what is the most important information concerning the patient that should be collected and transferred through the application? *Probe: basic information, contact info, health status, medications, complications, prior care received, etc.?*
24. How do you think that this technology would impact patient experience and information security? Can you provide an example? *Probe: shared facility phones, expectations around patient-provider interaction, monitoring of phone use, etc.*
25. Do you have any other ideas for how to use mobile technology and communication to connect women to the necessary health services?

Now we are going to talk about some possible ways that we might disseminate and use a mobile phone application to communicate between health providers and facilities on case management and patient transportation.

Section 6: Dissemination and Uptake Strategies

26. Thinking back to your workload timeline that we have created at the beginning of this session, how do you think this application would fit in your workday?
    a. How do you think using this application would change your chart?
    b. Do you have suggestions as to how this might be introduced into existing work and decision-making policies?
    c. If this application is introduced today, would you be willing to start using it? How do you think this might change the interaction with patients?

Please feel free to share any remaining ideas or reflections about the questions mentioned.

Section 7: Wrap-Up

27. How do you see technology and the use of mobile phone apps for supporting health being incorporated into daily health services in the next 5-10 years?
28. Do you have any other feedback or advice for the developers of this mobile app technology?
29. Are there any other issues or ideas that you would like to bring up? Any suggestions or feedback?

Thank you for your thoughts and time.
