# Supplementary material for: Stakeholders' Perspectives on the Challenges of Emergency Obstetric Referrals and the Feasibility and Acceptability of an mHealth Intervention in Northern Iraq
Source: Front Glob Womens Health. 2021 May 26;2:662256. doi: 10.3389/fgwh.2021.662256 (PMC8594019; doi:10.3389/fgwh.2021.662256)
Supplement: Supplementary file 4 [file Image_1.pdf]

Supplemental files – Photos

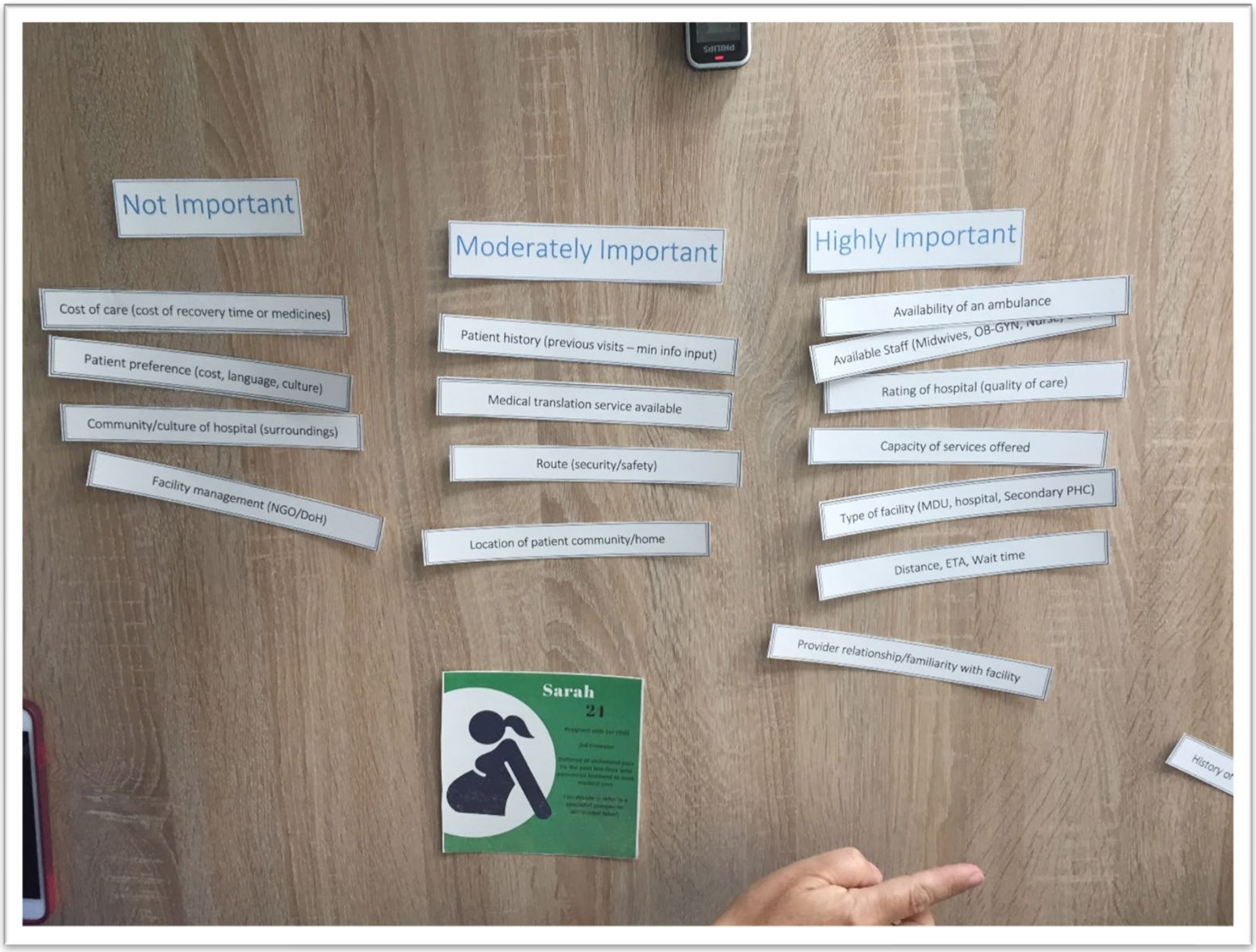

Figure 1: Referral Decision Making - Focus Group Discussion, Hospital Healthcare Personnel
